# Identification of four metabolic subtypes and key prognostic markers in lung adenocarcinoma based on glycolytic and glutaminolytic pathways

**DOI:** 10.1186/s12885-023-10622-x

**Published:** 2023-02-13

**Authors:** Jinjin Zhang, Xiaopeng Wang, Congkuan Song, Qi Li

**Affiliations:** 1 Department of Respiratory and Critical Care Medicine, Puren Hospital Affiliated to Wuhan Uiversity of Science and Technology, Wuhan, 430081 China; 2 Department of Thoracic Surgery, Renmin Hospital of Wuhan University, No.238 Jiefang Road, Wuchang District, Wuhan, 430060 China

**Keywords:** Lung adenocarcinoma (LUAD), Glycolysis, Glutaminolysis, Metabolic subtype

## Abstract

**Background:**

Glucose and glutamine are the main energy sources for tumor cells. Whether glycolysis and glutaminolysis play a critical role in driving the molecular subtypes of lung adenocarcinoma (LUAD) is unknown. This study attempts to identify LUAD metabolic subtypes with different characteristics and key genes based on gene transcription profiling data related to glycolysis and glutaminolysis, and to construct prognostic models to facilitate patient outcome prediction.

**Methods:**

LUAD related data were obtained from the Cancer Genome Atlas and Gene Expression Omnibus, including TCGA-LUAD, GSE42127, GSE68465, GSE72094, GSE29013, GSE31210, GSE30219, GSE37745, GSE50081. Unsupervised consensus clustering was used for the identification of LUAD subtypes. Differential expression analysis, weighted gene co-expression network analysis (WGCNA) and CytoNCA App in Cytoscape 3.9.0 were used for the screening of key genes. The Cox proportional hazards model was used for the construction of the prognostic risk model. Finally, qPCR analysis, immunohistochemistry and immunofluorescence colocalization were used to validate the core genes of the model.

**Result:**

This study identified four distinct characterized LUAD metabolic subtypes, glycolytic, glutaminolytic, mixed and quiescent types. The glycolytic type had a worse prognosis than the glutaminolytic type. Nine genes (CXCL8, CNR1, AGER, ALB, S100A7, SLC2A1, TH, SPP1, LEP) were identified as hub genes driving the glycolytic/glutaminolytic LUAD. In addition, the risk assessment model constructed based on three genes (SPP1, SLC2A1 and AGER) had good predictive performance and could be validated in multiple independent external LUAD cohorts. These three genes were differentially expressed in LUAD and lung normal tissues, and might be potential prognostic markers for LUAD.

**Conclusion:**

LUAD can be classified into four different characteristic metabolic subtypes based on the glycolysis- and glutaminolysis-related genes. Nine genes (CXCL8, CNR1, AGER, ALB, S100A7, SLC2A1, TH, SPP1, LEP) may play an important role in the subtype-intrinsic drive. This metabolic subtype classification, provides new biological insights into the previously established LUAD subtypes.

**Supplementary Information:**

The online version contains supplementary material available at 10.1186/s12885-023-10622-x.

## Introduction

Lung cancer remains the leading cancer type that threatens human life expectancy and quality of life worldwide [1]. Non-small cell lung cancer (NSCLC) is the predominant pathological type of all lung cancers, accounting for approximately 85% of cases, while lung adenocarcinoma (LUAD) is the most common subtype of NSCLC [2]. LUAD is characterized by a high rate of recurrence and metastasis. Despite appropriate surgery, chemotherapy, radiotherapy, targeted therapy, and immunotherapy, the 5-year survival of lung cancer patients is still only 16.8%, the prognosis of most LUAD patients remains suboptimal [3]. In addition, the complex pathogenesis of LUAD leads to inaccurate prediction of patient prognosis according to the current TNM staging system [4]. Therefore, it is urgent to decipher the tumorigenesis of LUAD and discover effective biomarkers and potential therapeutic targets to predict the prognosis of LUAD patients.

Cellular metabolic pathways mainly include glycolysis, lipid metabolism, glutaminolysis and oxidative phosphorylation [5]. The metabolism of cancer cells differs from that of normal cells in that they have elevated levels of metabolism and maintain a high proliferation rate that is used to resist some cell death signals [6]. This phenomenon is known as the “Warburg effect” and is closely related to the discovery of new therapeutic targets and the development of new anti-cancer drugs [7]. Angiogenesis is an important feature of tumor growth, but as the tumor grows, it causes some of the tumor tissues to become more distant from the blood vessels, so the content of glucose, oxygen and other components in these tissues decreases [8, 9]. Both glycolytic and glutaminolytic pathways are enhanced in cancer cells. Glycolysis can meet the energy requirements of cancer cells, and glutaminolysis can provide cancer cells with precursors for the synthesis of various substances. It has been found that the level of glucose in tumor extracellular fluid is half that of the surrounding normal tissue [10], a result that is consistent with some previous metabolomics studies reporting reduced glucose levels in malignant tissues [11]. Therefore, in rapidly proliferating and metastasis- and recurrence-prone tumors like LUAD, cancer cells are even more in need of rapid adaptation to glucose deficiency [12]. To adapt to this change, and to compensate for pyruvate, cancer cells maintain the citric acid cycle by accelerating the rate of glutamine catabolism. This series of reactions is of interest to cancer cells, as it can produce glutathione, fatty acids for the citric acid cycle, as well as carbon for nucleotides and even nitrogen for many non-essential amino acids [13]. Glutamine is the most abundant circulating amino acid in blood and muscle, and previous studies have found high intake of glutamine in many cancers including pancreatic, ovarian and breast cancers [14–16]. Glutamine is a key amino acid that supports many essential cellular functions in cancer cells, so glutaminolysis is closely related to the development of cancer cells [17].

**Fig. 1:**
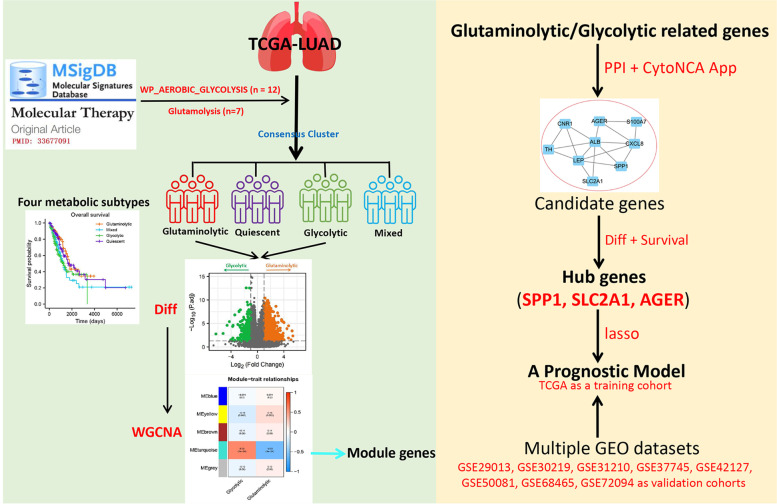
The detailed workflow of this study

Inhibition of key enzymes in the glycolytic and glutaminolytic pathways is emerging as a popular area of cancer research, and inhibition of these pathways has been shown to be effective in suppressing cancer cell proliferation [17–20]. The aim of this study was to identify LUAD metabolic subtypes with different prognosis. Patients were classified into different subtypes based on the expression of based on the glycolysis- and glutaminolysis-related genes. We explored the differences in clinical characteristics including prognosis of LUAD patients with different metabolic subtypes, and screened for possible prognostic markers in LUAD thus establishing a clinically feasible prognostic model that is expected to guide and design targeted therapies for LUAD in the future. The detailed workflow of this study was visible in Fig. 1.

## Materials and methods

### Data download and pre-processing

Five hundred ninety-five LUAD tissues and normal lung tissues, simple nucleotide variations (SNVs), and copy number variations (CNVs) were downloaded from the TCGA GDC portal (https://portal.gdc.cancer.gov/). Clinical data and survival data for LUAD were from UCSC Xena Portal. The gene of aerobic glycolytic pathways is derived from WP_AEROBIC_GLYCOLYSIS (*n* = 12) in the MSigDB database (https://www.gsea-msigdb.org/gsea/msigdb/index.jsp). The gene (*n* = 7) of glutaminolytic pathways from a previous study [21]. A total of 18 genes were included in the analysis excluding genes whose expression level was 0 in 50% of the samples. In addition, we also obtained some LUAD microchip datasets with detailed survival information and transcription profile data from the GEO database (https://www.ncbi.nlm.nih.gov/geo/). These LUAD datasets are GSE29013 [22], GSE30219 [23], GSE31210 [24, 25], GSE37745 [26–28], GSE42127 [29, 30], GSE50081 [31], GSE68465 [32], GSE72094 [33] respectively. We merged the datasets originating from the same platform (GSE29013, GSE30219, GSE31210, GSE37745, GSE50081), and we used the ‘ComBat’ algorithm of ‘sva’ R package to reduce the batch effect due to the non-biotechnical bias [34].

### Identification of different metabolic subtypes by consensus clustering

Based on the above 18 genes of the aerobic glycolytic pathway and glutaminolytic pathway, we performed consensus clustering of LUAD samples using the “ConsensusClusterPlus” R package. We refer to previous studies to perform specific practices [35–37]. Subsequently, we classified the LUAD samples into different metabolic subtypes based on the median of the two types of co-expressed genes. They were glycolytic type (glycolytic median > 0 and glutaminolytic median < = 0), glutaminolytic type (glycolytic median < = 0 and glutaminolytic median > 0), mixed type (glycolytic median > 0 and glutaminolytic median > 0) and quiescent type (glycolytic median < 0 and glutaminolytic median < 0).

### Weighted gene co-expression network analysis (WGCNA) and functional annotations

We explored the genes differentially expressed between glycolytic type and glutaminolytic type tumor tissues using the limma R package. The threshold was set as:|log2FC| > 1 and FDR < 0.05. Differential expressions were presented in the form of volcano maps. Subsequently, a weighted gene co-expression network analysis (WGCNA) of the above differential genes was performed using the WGCNA R package. Accordingly, the correlation between each gene was obtained, and the correlation matrix and topological overlap matrices between the genes were constructed to measure the network connectivity of the genes and to determine the soft threshold size. Genes with similar expression levels were grouped into a gene module with linkage hierarchical clustering. The weight of each module in the dataset was also calculated, and the maximum weight dataset was filtered for subsequent analysis. Subsequently, we selected the modules most related to the glycolytic and glutaminolytic types, and functionally annotated this module genes using the Metascape website (https://metascape.org/gp/index.html).

### Screening of hub genes associated to glycolytic\glutaminolytic type

The genes within the significance module obtained above were imported into the STRING database (https://cn.string-db.org/) to construct a protein-protein interaction (PPI) network, which was also visualized by using Cyctoscape software. The minimum interaction score > 0.5 between proteins was selected in the setup as the basis for the reliability of protein interactions, and the nodes outside the network linkage were hidden to finally obtain the grid map of protein interactions. The topological analysis of the PPI network was performed by CytoNCA app, using “Betweenness”, “Closeness”, “Degree”, “Eigenvector”, “LAC” and “Network” as standard reference, and the genes whose all standard scores were greater than their corresponding standard mean were selected as candidate targets, which were then subjected to the above topological analysis twice to obtain the candidate genes. Subsequently, we further explored the differential expression of these genes between tumor and normal tissues and the relationship with overall survival of patients. Next, we identified the genes that were differentially expressed between LUAD and normal lung tissues and significantly correlated with prognosis as Hub genes.

### Construction and validation of a hub gene-based prognostic model

Here, a more prevalent machine learning algorithm - LASSO was used to construct a Hub gene-based prognostic model for LUAD (TCGA-LUAD, *n* = 500). The relevant parameters in this process were as follows: family = “cox”, maxit = 1200, and other parameters were set as the default values. The risk score for each patient was obtained by the following formula: Riskscore = CoefG1*Expression***G1*** + CoefG2*Expression***G2*** + ... + CoefGn*Expression***Gn***. Using the median value of the risk score, patients were divided into two groups. and Kaplan-Meier survival analysis was performed to verify the effectiveness of this model in the training cohort (n = 500) and multiple independent validation cohorts, including GSE42127 cohort (*n* = 133), GSE68465 cohort (*n* = 442), GSE72094 cohort (*n* = 398), and merge-GEO cohort (GSE29013, GSE30219, GSE31210, GSE37745, GSE50081) (*n* = 574).

### Cell culture and quantitative real-time polymerase chain reaction (qRT-PCR)

Lung cancer cell lines (H1299 and A549) and normal lung epithelial cell line (Beas-2B) were purchased from the American Type Culture Collection (ATCC, Manassas, VA). H1299 and A549 cells were cultured using RPMI 1640 medium, and Beas-2B cells were cultured in DMEM medium. These mediums contain 10% fetal bovine serum (FBS), 50 mg/mLstreptomycin and 50 IU/mL penicillin, and all cells were placed in an incubator conditioned at 37 °C, 5% CO2. All cell lines were tested and authenticated by short tandem repeat (STR) analysis. The Hub gene mRNA expression in cells was measured using qRT-PCR analysis. Applying the Trizol method, the Total RNA was extracted and subsequently used to synthesize cDNA and subjected to PCR reactions (all experimental procedures were performed strictly according to the instructions of the kit). The primer sequences of Hub genes, and GAPDH used in the RT–qPCR are listed in Table S1.GAPDH was used as the reference gene and relative gene expression was calculated by the 2 − ΔΔCT method.

### Immunohistochemistry (IHC) and subcellular localization of the proteins encoded by the hub genes

In the above analysis, we identified the Hub genes. To further explore the expression levels of the Hub gene-encoded proteins in LUAD tumor tissues and normal lung tissues, we selected their immunohistochemical results for presentation. These IHC results can be obtained from an open-source database (The Human Protein Atlas (HPA): https://www.proteinatlas.org/). Subsequently, we were interested in the subcellular localization of their proteins, and for this purpose, we also obtained immunofluorescence and confocal images of the subcellular localization of the Hub genes in cancer cells (Hep G2) from this database.

## Results

### Identification of the four metabolic subtypes of LUAD

**Fig. 2:**
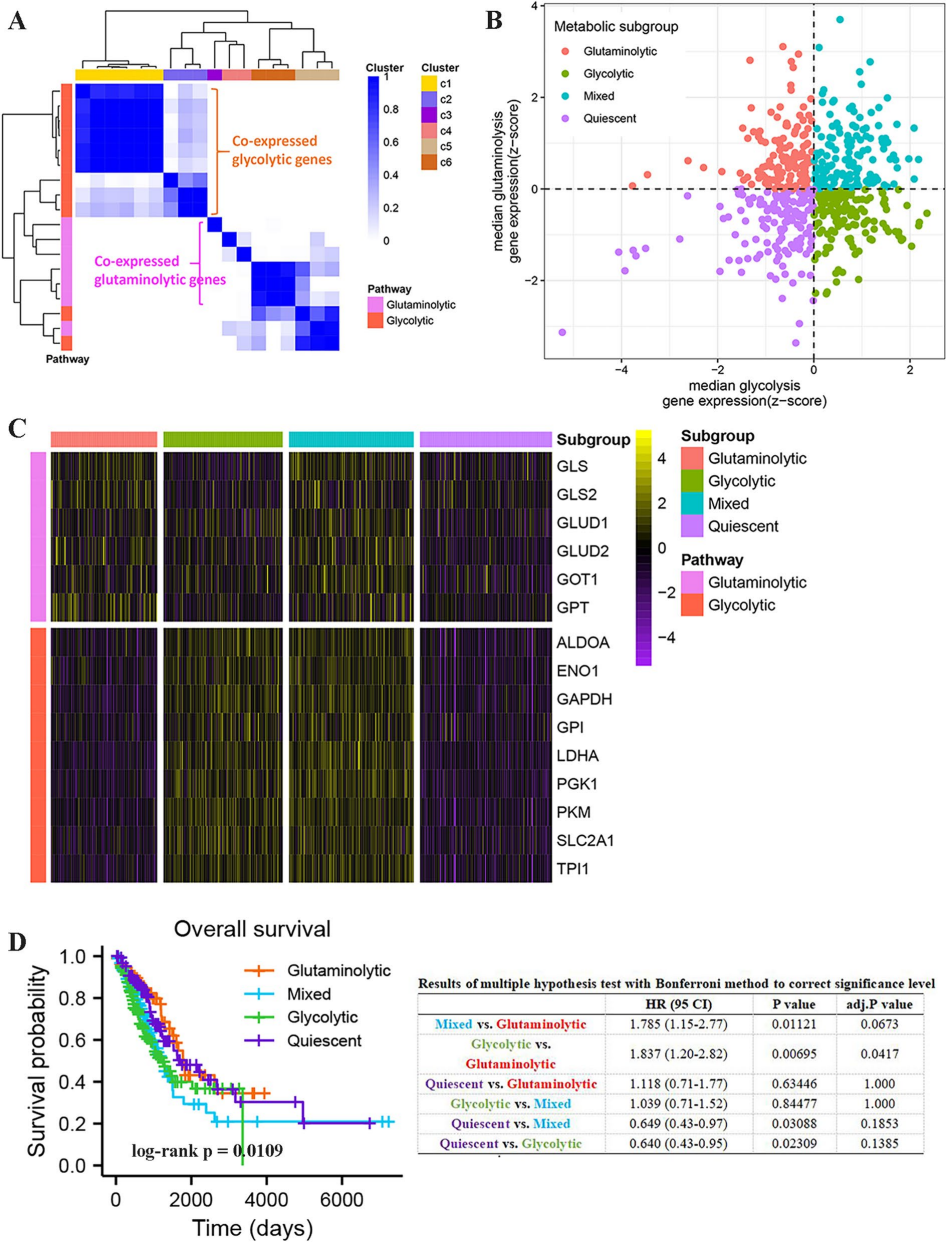
Identification of the four metabolic subtypes of LUAD. **A** Consistent clustering of the aerobic glycolytic and glutaminolytic pathway related genes. **B** The four LUAD metabolic subtypes (Glycolytic, Glutaminolytic, Quiescent, and Mixed) were identified according to aerobic glycolytic and glutaminolytic pathway related gene expression levels. **C** The heatmap showing the expression levels of these selected genes in the four subgroups. **D** Overall survival time prognostic survival curves of the four molecular subtypes

Consensus clustering of 11 genes of the glycolytic pathway and 7 genes of the glutaminolytic pathway was performed using TCGA-LUAD data to screen for co-expression of glycolytic and glutaminolytic related genes. When K = 6, glycolytic and glutaminolytic genes were clustered together. As shown in Fig. 2a, the genes in C1 and C2 (defined as glycolytic co-expression genes) all belong to the glycolytic pathway, and these genes include ALDOA, ENO1, GAPDH, GPI, PKM, TPI1, LDHA, PGK1, SLC2A1. The genes in C3, C4 and C6 (defined as glutaminolytic co-expression genes) all belong to the glutaminolytic pathway, and these genes include GLS, GOT1, GPT, GLS2, GLUD1, and GLUD2. Subsequently, the median expression values of co-expressed glycolytic and glutaminolytic genes were z-scored, and four metabolic subgroups were identified based on median expression (Fig. 2b). The expression levels of these selected genes were visualized in the four subgroups, with glutaminolytic genes being generally highly expressed in the glutaminolytic and mixed types but relatively low in the Quiescent and glycolytic types. Glycolytic genes were highly expressed in the glycolytic and mixed types, but low in the Quiescent and glutaminolytic types (Fig. 2c). Next, we investigated the prognostic relationships among these four LUAD subtypes, and the results of Kaplan-Meier survival analysis indicated that OS was significantly different among the four subtypes. Even with multiple hypothesis tests with the Bonferroni method to correct the significance level, the overall survival of glycolytic patients remained significantly worse than the glutaminolytic patients (Fig. 2d). In addition, we also analyzed the distribution of clinical characteristics among the four different subtypes. Extensive similarities in clinical features exist among patients of the four metabolic subtypes, however, they differ in the M stage as well as in the anatomic neoplasm subdivision, as shown in Table 1.

**Table 1:** Distribution and comparison of clinical characteristics across the four LUAD metabolic subtypes

Variables	Glycolytic (***n*** = 120)	Glutaminolytic (***n*** = 104)	Mixed (***n*** = 127)	Quiescent (***n*** = 136)	chi-square	***P*** value
**Age**					3.866	0.276
< = 60	41(34.17%)	24(23.08%)	41(32.28%)	45(33.09%)		
> 60	76(63.33%)	77(74.04%)	85(66.93%)	88(64.71%)		
NA	3(2.5%)	3(2.88%)	1(0.79%)	3(2.21%)		
**Sex**					0.803	0.849
Female	66(55%)	53(50.96%)	72(56.69%)	73(53.68%)		
Male	54(45%)	51(49.04%)	55(43.31%)	63(46.32%)		
**T_stage**					0.513	0.916
T1&T2	105(87.5%)	92(88.46%)	109(85.83%)	116(85.29%)		
T3&T4	14(11.67%)	12(11.54%)	17(13.39%)	19(14.96%)		
Tx	1(0.83%)	0(0)	1(0.79%)	1(0.79%)		
**N_stage**					4.065	0.255
N0	84(70%)	69(66.35%)	79(62.20%)	81(59.56%)		
N1&N2%N3	33(27.5%)	33(31.73%)	46(36.22%)	53(38.97%)		
Nx	3(2.5%)	2(1.92%)	2(1.57%)	2(1.47%)		
**M_stage**					9.078	0.028
M0	82(68.33%)	66(63.46%)	85(66.93%)	89(65.44%)		
M1	2(1.67%)	2(1.92%)	12(9.45%)	8(5.88%)		
Mx	36(30%)	36(34.62%)	30(23.62%)	39(28.68%)		
**TNM_stage**					4.006	0.261
I&II	97(80.8%)	84(80.77%)	94(74.01%)	99(72.79%)		
III&IV	20(16.7%)	20(19.23%)	29(22.83%)	36(26.47%)		
NA	3(2.5%)	0	4(3.15%)	1(0.74%)		
**New_Event_Type**					9.835	0.132
Distant Metastasis	13(10.83%)	27(25.96%)	16(12.60%)	16(11.76%)		
Locoregional Recurrence	18(15%)	9(8.65%)	15(11.81%)	14(10.29%)		
New Primary Tumor	3(2.5%)	3(2.88%)	1(0.79%)	4(2.94%)		
NA	86(71.67%)	75(72.12%)	95(74.80%)	102(75%)		
**Anatomic_neoplasm_subdivision**					28.414	0.005
R-Lower	18(15%)	17(16.35%)	31(24.41%)	27(19.85%)		
R-Middle	6(5%)	9(8.65%)	5(3.94%)	1(0.74%)		
R-Upper	35(29.17%)	36(34.62%)	46(36.22%)	53(38.97%)		
L-Lower	25(20.83%)	13(12.5%)	23(18.11%)	14(10.29%)		
L-Upper	33(27.5%)	27(25.96%)	16(12.60%)	38(27.94%)		
Others/NA	3(2.5%)	2(1.92%)	6(4.72%)	3(2.21%)		
**location_in_lung_parenchyma**					2.889	0.409
Peripheral Lung	29(24.17%)	30(28.85%)	28(22.05%)	31(22.79%)		
Central Lung	11(9.17%)	12(11.54%)	17(13.39%)	22(16.18%)		
NA	80(66.67%)	62(59.62%)	82(64.57%)	83(61.03%)		

### Identification of hub genes associated with glycolytic/glutaminolytic type

**Fig. 3:**
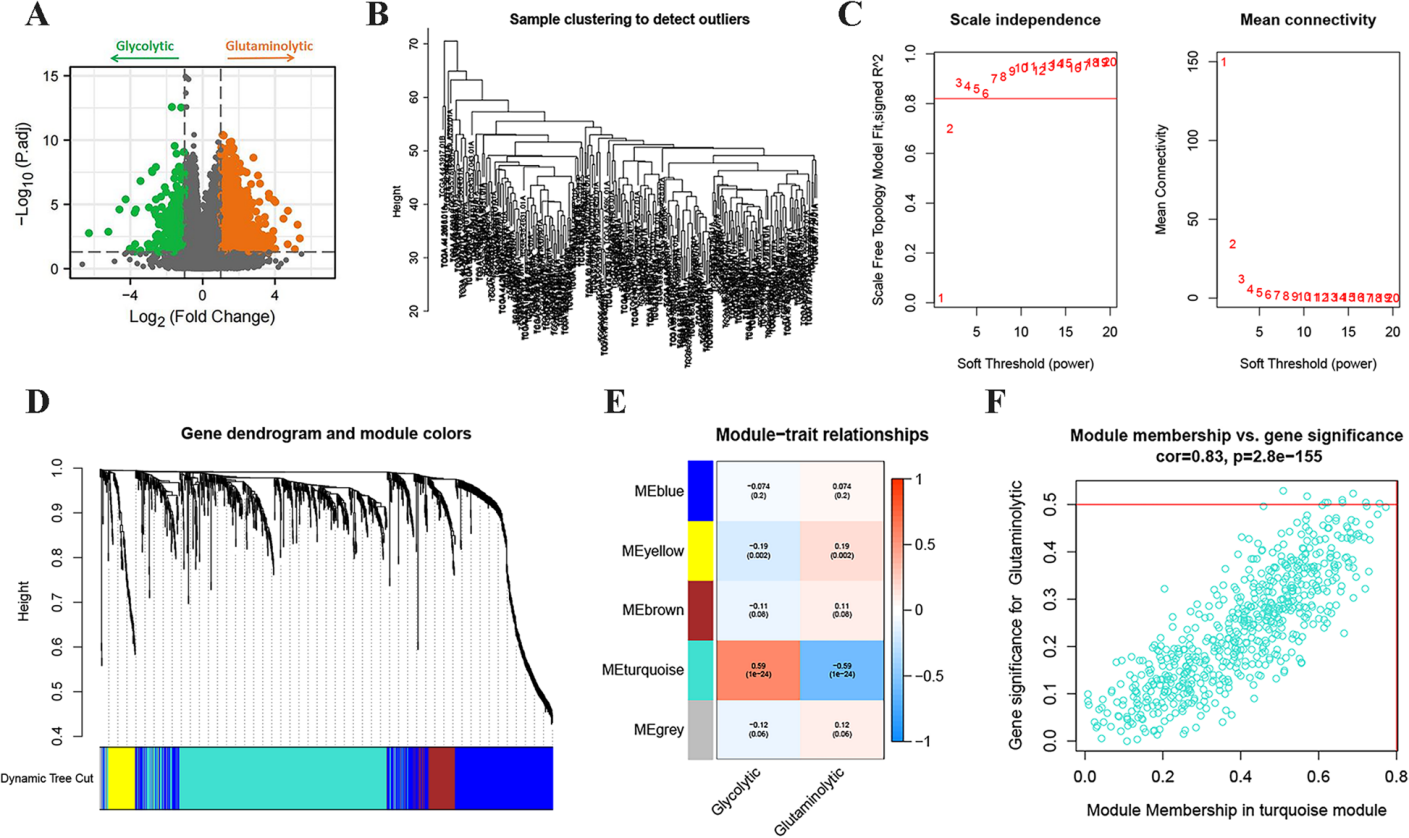
Differentially expressed genes and WGCNA between glycolytic and glutaminolytic samples. **A** The Volcano plots showing the distribution of differentially expressed genes between the two subtypes. **B** Cluster plot of the LUAD samples. **C** Screening of the optimal soft-threshold values. **D** Dendrogram of LUAD gene clustering. **E** Heatmap of the correlation of gene modules with the glycolytic / glutaminolytic type. **F** Correlation of the selected module membership with gene significance

**Fig. 4:**
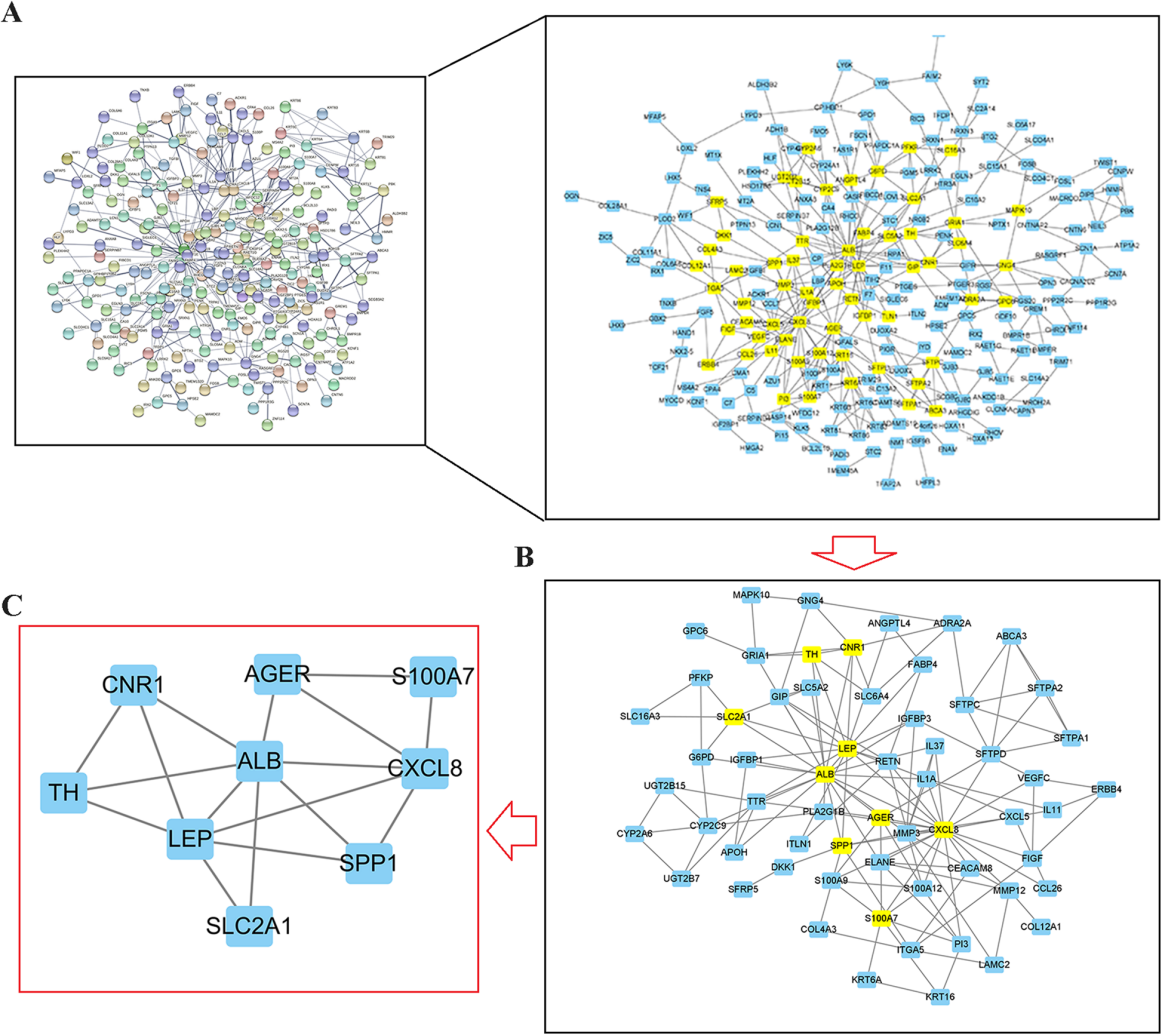
The PPI network and the topology analysis based on CytoNCA App in Cytoscape 3.9.0. **A** The PPI for the differentially expressed genes between the glycolytic and glutaminolytic samples. **B-C** The screening of candidate genes by two topological analysis

**Fig. 5:**
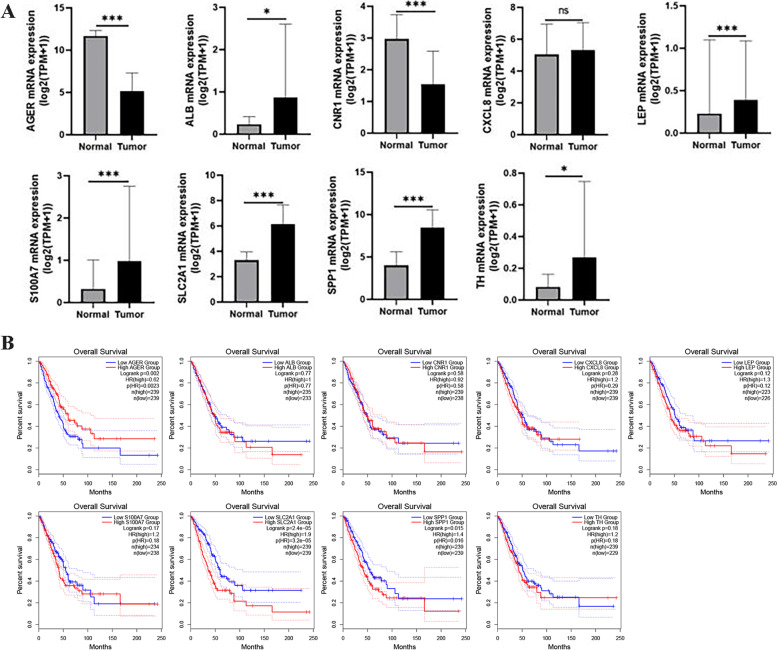
Expression of the candidate genes and their relationship to the overall survival. **A** Comparison of the expression of the 11 candidate genes between LUAD tumor and normal tissues. **B** Effect of 11 candidate genes on LUAD overall survival

In the previous analysis, we found significantly different prognostic differences between patients with glycolytic and glutaminolytic types. For this reason, we further investigated the Hub genes significantly associated with the two subtypes. First, we performed differential expression analysis of the transcriptional profiles of the two subtypes (Fig. 3a) and obtained a total of 2772 differentially expressed genes, including 319 genes upregulated in the glycolytic type and 2453 genes upregulated in the glutaminolytic type. We performed WGCNA analysis of glycolytic and glutaminolytic types of samples based on these 2772 differentially expressed genes. To remove sample outliers, we clustered the LUAD glycolytic and glutaminolytic types of samples based on the gene expression matrix and constructed a clustering dendrogram, where the ordinate indicates each sample and the abscissa indicate the clustering distance. The results showed that none of these samples were significantly deviated, and no samples were rejected (Fig. 3b). We set the selection criterion of soft threshold as signed R^2^ > 0.82, selected a set of candidate thresholds and output the corresponding network parameters, as in Fig. 3c, when the soft threshold was 6, the gene network satisfies both high internal connectivity and high gene similarity. The gene co-expression network was constructed with a threshold value of 6, and the differentially expressed genes were clustered hierarchically according to the dissimilarity matrix, and a clustering dendrogram was constructed (Fig. 3d). The network modules were set to contain at least 50 genes, and the different gene modules were identified using the dynamic cut method, and the modules with high similarity were merged to finally obtain five different gene modules. These different gene modules were indicated by different colors, and the genes in the same color module had high similarity. To screen the modules with high correlation with LUAD glycolytic and glutaminolytic types, we first did principal component analysis (PCA) on the genes in each module separately, extracted the value of the first principal component as the module eigenvalue (ME), and then calculated the correlation coefficient between the ME and glycolytic\glutaminolytic type, and the correlation heat map was shown in Fig. 3e. We found that the turquoise color module was most correlated with glycolysis\glutaminolytic type. The genetic significance of the turquoise color module and the module membership relationships were shown in Fig. 3f. The values of these variables showed a strong positive correlation (cor = 0.83, *p* = 2.8e-155). In view of the above, we conductd the turquoise color module to Metascape analysis (Table 2). The results showed that these genes are mainly involved in biological processes such as naba matrisome-associated, response to hypoxia, regulation of hormone levels, and response to extracellular stimulus. Subsequently, in order to further screen out the genes related to glycolytic\glutaminolytic type, we input the above turquoise module genes into String database, selected human genes for PPI network analysis (related protein nodes were filtered by protein interaction score ≥ 0.5 criterion), the PPI network TSV format file was imported into Cytoscape 3.9.0 to obtain the PPI network map (Fig. 4a), and the PPI network was topologically analyzed twice by CytoNCA app (Fig. 4b-c), and finally nine candidate genes (CXCL8, CNR1, AGER, ALB, S100A7, SLC2A1, TH, SPP1, LEP) were obtained. Further exploring the expression levels of these nine genes between LUAD tumors and normal tissues and their relationship with prognosis (Fig. 5), we found that only three genes (SPP1, SLC2A1 and AGER) were both differentially expressed and associated with prognosis. Thus, they were identified as glycolytic\glutaminolytic-related Hub genes.

**Table 2:** The metascape analysis of the module genes

GO	Category	Description	Count	%	Log10(P)	Log10(q)
M5885	Canonical Pathways	NABA MATRISOME ASSOCIATED	55	12.67	−22.49	−18.14
GO:0001666	GO Biological Processes	response to hypoxia	23	5.3	−10.73	−6.85
GO:0010817	GO Biological Processes	regulation of hormone levels	28	6.45	−8.95	−5.38
GO:0009991	GO Biological Processes	response to extracellular stimulus	27	6.22	−8.67	−5.17
GO:0031424	GO Biological Processes	keratinization	12	2.76	−8.59	−5.14
GO:0048729	GO Biological Processes	tissue morphogenesis	29	6.68	−8.49	−5.09
GO:0019730	GO Biological Processes	antimicrobial humoral response	13	3	−8.06	−4.76
WP2882	WikiPathways	Nuclear receptors meta-pathway	21	4.84	−7.95	−4.76
GO:0045766	GO Biological Processes	positive regulation of angiogenesis	16	3.69	−7.93	−4.76
GO:0032103	GO Biological Processes	positive regulation of response to external stimulus	25	5.76	−7.85	−4.71
R-HSA-1474244	Reactome Gene Sets	Extracellular matrix organization	20	4.61	−7.74	−4.62
GO:0006935	GO Biological Processes	chemotaxis	26	5.99	−7.55	−4.48
GO:0050927	GO Biological Processes	positive regulation of positive chemotaxis	7	1.61	−7.33	−4.31
R-HSA-5687613	Reactome Gene Sets	Diseases associated with surfactant metabolism	5	1.15	−7.14	−4.16
M5884	Canonical Pathways	NABA CORE MATRISOME	18	4.15	−6.93	−4.03
WP2877	WikiPathways	Vitamin D receptor pathway	14	3.23	−6.24	−3.51
GO:0055082	GO Biological Processes	cellular chemical homeostasis	24	5.53	−6.16	−3.43
GO:0044703	GO Biological Processes	multi-organism reproductive process	14	3.23	−6.13	−3.42
hsa05133	KEGG Pathway	Pertussis	9	2.07	−5.83	−3.25
WP2806	WikiPathways	Complement system	10	2.3	−5.77	−3.2

### Estimation of a prognostic model

**Fig. 6:**
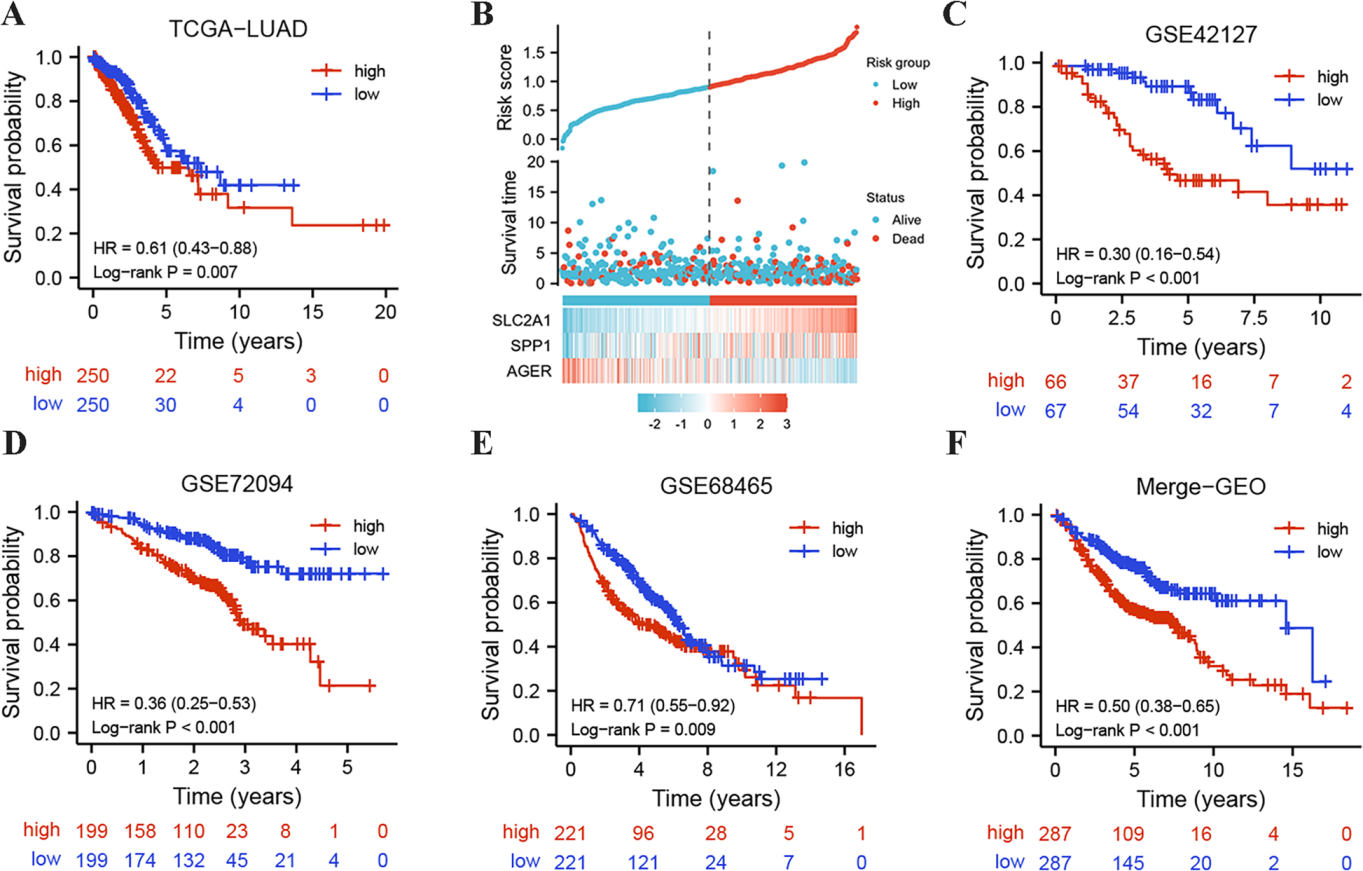
Validation of the LUAD risk assessment model. **A** Kaplan-Meier survival curves for patients at high and low risk in TCGA. **B** Patients of TCGA-LUAD were arranged in the same ascending order of the risk score. **C** Kaplan-Meier survival curves for patients at high and low risk in GSE42127. **D** Kaplan-Meier survival curves for patients at high and low risk in GSE72094. **E** Kaplan-Meier survival curves for patients at high and low risk in GSE68465. **F** Kaplan-Meier survival curves for patients at high and low risk in merge-GEO (GSE29013, GSE31210, GSE30219, GSE37745, GSE50081)

In the previous analysis, we identified 3 Hub genes that were closely associated with the glycolytic\glutaminolytic types. Considering their significant impact in terms of patient outcome (they were also considered as prognostic markers of LUAD), we constructed a risk model that can be used to assess the prognosis of LUAD patients based on the transcript expression profiles of these 3 genes using the LASSO method. The risk score for each patient was calculated by the following formula: Riskscore = 0.00236992044579479 * Expression (SPP1) + 0.205240153500597 * Expression (SLC2A1) + (− 0.0441022899873212) * Expression (AGER). Using the median of the patients’ risk scores as the cut-off point, patients were divided into high-risk and low-risk groups. In the TCGA cohort, we can clearly find that the prognosis of patients with high risk score was significantly worse than that of the low risk group (Fig. 6a). Patient survival status and Hub gene expression changed with risk score, more patients died in the high-risk group and the expression of SPP1 and SLC2A1 increased with increasing risk score, while the opposite was true for AGER (Fig. 6b). In addition, independent prognostic analysis also suggested that the risk score could also be used to assess patient prognosis independently of other factors (Table 3). To further validate the stability of the model, we also performed validation in several iindependent external validation cohorts, including the GSE42127 cohort (Fig. 6c), the GSE72094 cohort (Fig. 6d), the GSE68465 cohort (Fig. 6e), and the Meta-GEO cohort (Fig. 6f). Overall, these results suggested that the prognostic model constructed in this study can predict patient outcomes more consistently and accurately.

**Table 3:** Independent prognostic analysis based on clinical characteristics and risk score

Variables	Univariate analysis		Multivariate analysis	
	HR(95% CI)	*p* value	HR(95% CI)	*p* value
**Age**	1.012(0.993–1.032)	0.213		
**Sex** (Male vs Female)	0.916(0.636–1.321)	0.639		
**T_stage** (T3/T4 vs T1/T2)	2.119(1.315–3.413)	0.002	1.273(0.745–2.175)	0.377
**N_stage** (N1–3 vs N0)	3.161(2.186–4.569)	9.30644E-10	2.248(1.445–3.493)	0.0003
**M_stage** (M1 vs M0)	2.028(1.051–3.911)	0.035	1.436(0.690–2.990)	0.333
**M_stage** (Unk vs M0)	0.586(0.359–0.953)	0.031	0.699(0.427–1.145)	0.155
**TNM_Stage** (III/IV vs I/II)	3.054(2.099–4.445)	5.49327E-09	1.456(0.862–2.461)	0.160
**riskScore**	3.752(2.087–6.748)	1.00289E-05	2.336(1.285–4.246)	0.0005

### SPP1, SLC2A1, AGER as key prognostic markers for LUAD

**Fig. 7:**
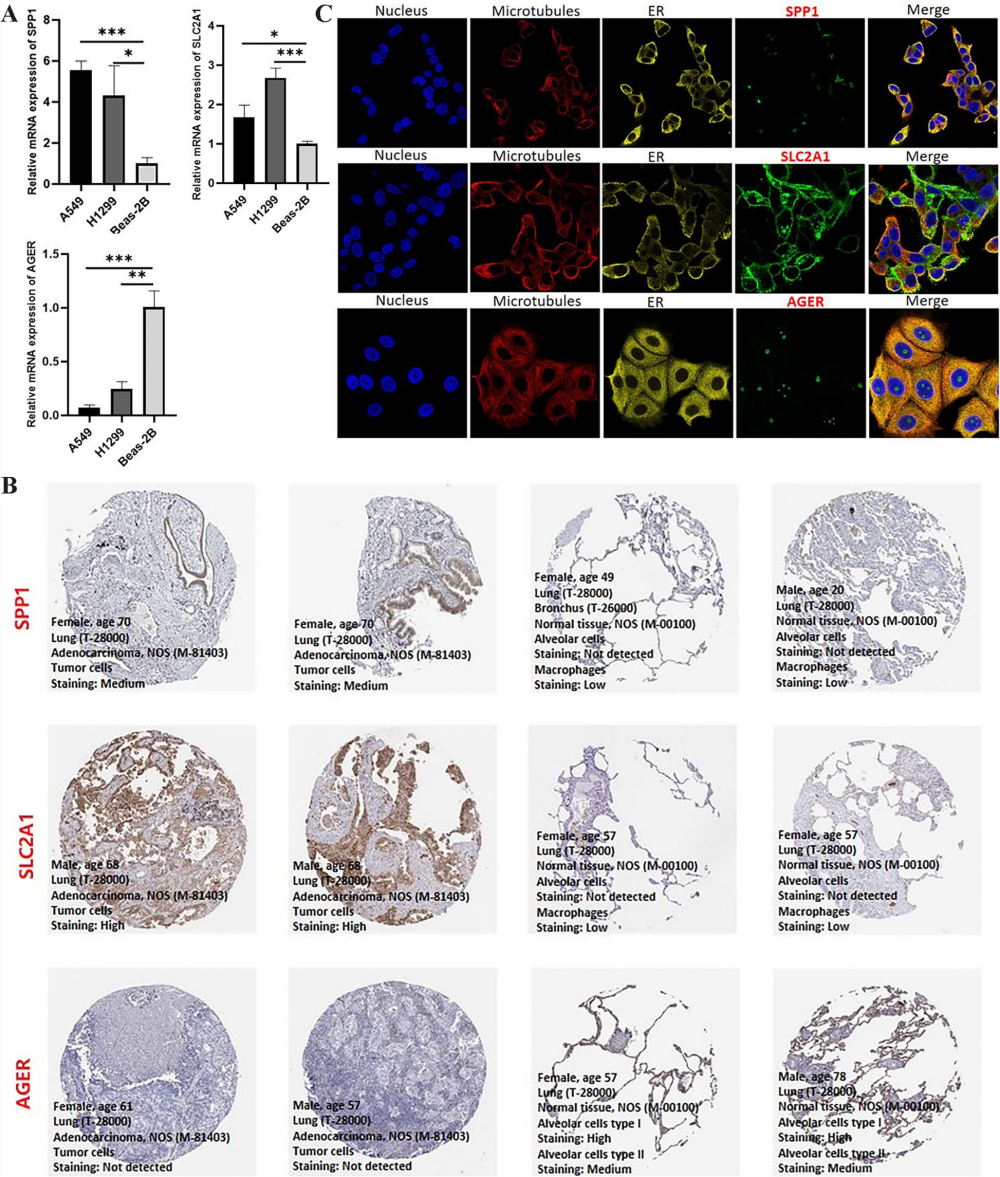
The qPCR, immunofluorescence and confocal microscopy, and immunohistochemistry of the hub genes. **A** The qPCR revealed the expression of three genes in two LUAD cell lines and normal lung epithelial cells. **B** The immunohistochemistry of the hub genes in LUAD tissues and normal lung tissues. **C** Immunofluorescence and confocal microscopy of the hub genes in human cancer cells (Hep G2)

In the previous analysis, we identified 3 LUAD prognostic markers, and their expression levels in LUAD were initially revealed by bioinformatic methods. To further clarify this relationship, we performed qPCR analysis in human normal lung epithelial cell line (Beas-2B) and lung cancer cell lines (H1299 and A549) (Fig. 7a). The results showed that SPP1, SLC2A1 was significantly highly expressed in the lung cancer cell, while AGER was in the opposite. These results were consistent with the above findings. In addition, we also verified their expression in LUAD tumors and normal tissues from the protein level (Fig. 7b). Further, we used images obtained by immunofluorescence and confocal microscopy to point out the subcellular localization profiles of the proteins of these three prognostic markers in human cancer cells (Fig. 7c). These findings were beneficial to improve the understanding of these three prognostic markers.

## Discussion

As one of the more heterogeneous malignant tumors, LUAD has its complex oncogenic mechanism [38]. Although the current study has largely increased awareness of LUAD, the treatments for LUAD are significantly diversified, and the prognosis is significantly improved, it is undeniable that there is still a large room for improvement in the prognosis of LUAD. Therefore, it is imperative to further refine the LUAD research and accelerate the individualized treatment of LUAD.

Glucose and glutamine are the main energy sources of tumor cells, and the metabolic abnormalities of both substances significantly affect the tumor cell fate. In view of this, we attempted to classify LUAD patients into differently characterized tumor subtypes based on the transcriptional profile data of the two metabolic genes involved in glycolysis and glutaminolysis. Our results showed that LUAD could be divided into four different metabolic subtypes: Glycolytic, Glutaminolytic, Mixed, and Quiescent. Overall survival remained significantly worse in glycolytic type patients than in glutaminolytic ones. In univariate analysis, we observed that M stage has a large impact on patient prognosis and is a poor prognostic factor. That could be expected. Also, the proportion of M1 patients varies among the different subtypes. Among them, M1 had the highest proportion of Mixed type at 9.45%. This also seems to correspond to a poor prognosis of the Mixed subtype. Interestingly, the glutaminolytic type had a significantly different prognosis from the glycolytic type, however, both did not differ significantly different in M stage. This further emphasizes the deficiency of M stage in the prognostic stratification of LUAD patients, and that the more rational molecular typing of LUAD can still be mined. This illustrates to some extent the importance of the four LUAD metabolic subtypes identified in this study. It is noteworthy that the proportion of patients with M1 was too small in the LUAD sample, which may also be an important factor influencing the prognostic stratification.

Furthermore, our study also identified nine hub genes closely related to the glycolytic/ glutaminolytic LUAD (CXCL8, CNR1, AGER, ALB, S100A7, SLC2A1, TH, SPP1, LEP), which were also further filtered for significant markers affecting LUAD prognosis, thus constructing a risk assessment model. Three key genes (SPP1, SLC2A1 and AGER) were selected for the model construction. The results showed that this risk prognostic model robustly divided LUAD into subgroups with significantly different prognosis, and that this accuracy was also validated in multiple LUAD cohorts. SPP1 is a secreted multifunctional phosphoprotein, also known as bone bridge protein-like protein or early T lymphocyte activation 1 protein, that specifically binds and activates matrix metalloproteinases (MMPs) in cancer [39]. Its main function is to participate in immune response and tissue remodeling, and it is also associated with cell growth, proliferation, migration, and apoptosis [40]. Previous findings have found that SPP1 shows high expression in many cancers and can be used to predict patient prognosis, including ovarian cancer, glioblastoma, hepatocellular carcinoma and gastric cancer [41–43], but no studies have been shown to explore the relevance of SPP1 to LUAD,therefore our study may prove to SPP1 in LUAD and its potential clinical value. Among the many genes associated with glucose metabolism, SLC2A1 is the gene encoding a glucose transporter protein that controls glucose uptake and plays a key role in the growth and proliferation of tumor cells [44, 45]. SLC2A1 has been reported to be aberrantly expressed in several cancer types and is closely associated with the development and progression of human cancer [46–48]. SLC2A1 was found to be significantly overexpressed in LUAD and closely correlated with overall survival (OS) of patients, which is consistent with the results we obtained. AGER is a highly polymorphic gene with polymorphisms or SNPs that may be responsible or co-responsible for disease development, is expressed primarily in the lung, and is involved in multiple pathways that initiate and maintain an unfavorable pro-inflammatory state [49]. AGER overexpression decreased proliferation, invasion and migration of LUAD cells H1299 and increased apoptosis, AGER may act as a potential molecular marker for LUAD [50], this is very much in line with our expections. Interestingly, another study found that blocking AGER could inhibit cervical squamous cell proliferation and migration, while overexpression of AGER could increase cell proliferation and migration, and inhibit cell apoptosis [51].

Although our study utilized powerful open-source data information to reveal four different features of LUAD metabolic subtypes and to construct a robust risk assessment model, some limitations of this study remain. First, the intrinsic molecular driving mechanisms of the four metabolic isoforms identified in this study were not explored by the underlying experiments. Secondly, the prognostic model we developed was based only on the transcriptional profile information of key genes, and other omics information, such as genome and proteomics information, was lacking. Our prognostic model can refer to more data features at follow-up to further improve the accuracy of the prognostic model.

Overall, our study is the first to identify four distinct LUAD metabolic isotypes based on gene transcription profiling data related to glycolysis and glutaminysis. Nine genes (CXCL8, CNR1, AGER, ALB, S100A7, SLC2A1, TH, SPP1, LEP) may play an important role in the subtype-intrinsic drive. We explored the differences in survival and other clinical characteristics of LUAD patients with different metabolic subtypes, and screened potential prognostic markers in LUAD, thus establishing a clinically feasible prognostic model that is expected to guide and design future targeted therapies for LUAD.

## Supplementary Information

**Additional file 1: Table S1.** Primer information for the three Hub genes and the reference genes.

## Data Availability

The datasets generated and analysed during the current study are available in the TCGA GDC repository, (https://portal.gdc.cancer.gov), GEO repository, (https://www.ncbi.nlm.nih.gov/geo/), Human Protein Atlas (HPA) database (http://www.proteinatlas.org/).
