# Supplementary material for: Identification of four metabolic subtypes and key prognostic markers in lung adenocarcinoma based on glycolytic and glutaminolytic pathways
Source: BMC Cancer. 2023 Feb 13;23:152. doi: 10.1186/s12885-023-10622-x (PMC9926575; doi:10.1186/s12885-023-10622-x)
Supplement: Supplementary file 1 — Additional file 1: Table S1. Primer information for the three Hub genes and the reference genes. [file 12885_2023_10622_MOESM1_ESM.docx]

Table S1. Primer information for the three Hub genes and the reference genes.

| **Primer name** | **Primer sequences（5'-3')** | **Fragment length（bp）** | **Annealing temperature （℃）** | **GC%** | **Base number** | **nmol/OD** |
| --- | --- | --- | --- | --- | --- | --- |
| H-GAPDH-S | GGAAGCTTGTCATCAATGGAAATC | 168 | 62.4 | 57.1 | 24 | 4.16 |
| H-GAPDH-A | TGATGACCCTTTTGGCTCCC |  | 62 | 55 | 20 | 5.79 |
|  |  |  |  |  |  |  |
| H-SPP1-S | AGCTTTACAACAAATACCCAGATGC | 98 | 54.3 | 40 | 25 | 3.51 |
| H-SPP1-A | CTTACTTGGAAGGGTCTGTGG |  | 54.2 | 52.38 | 21 | 4.47 |
|  |  |  |  |  |  |  |
| H-SLC2A1(1)-S | GCTTCTCCAACTGGACCTCAAA | 113 | 53.7 | 50 | 22 | 4.26 |
| H-SLC2A1(1)-A | GAAGAACAGAACCAGGAGCACAG |  | 55.9 | 52.17 | 23 | 3.53 |
|  |  |  |  |  |  |  |
| H-AGER（1）-S | CTCAGGACCAGGGAACCTACAG | 220 | 58.4 | 59.09 | 22 | 4.05 |
| H-AGER（1）-A | CGCCTTTGCCACAAGATGAC |  | 53.7 | 55 | 20 | 4.75 |
